# Variation in fine root traits with thinning intensity in a Chinese fir plantation insights from branching order and functional groups

**DOI:** 10.1038/s41598-021-02206-1

**Published:** 2021-11-22

**Authors:** Zuhua Wang, Min Liu, Fen Chen, Haibo Li

**Affiliations:** 1 College of A&F Engineering and Planning, Tongren University, TongrenGuizhou, 554300 China; 2 National Nature Reserve Administration of the Fanjing Mountain, TongrenGuizhou, 554300 China

**Keywords:** Forestry, Forest ecology

## Abstract

Thinning is a widely used practice in forest management, but the acclimation mechanisms of fine roots to forest thinning are still unclear. We examined the variations in fine root traits of different branching orders and functional groups along a thinning intensity gradient in a 26-year-old Chinese fir (*Cunninghamia lanceolata*) plantation. With increasing thinning intensity, the root C concentration (RCC), root N concentration (RNC), specific root area (SRA), and specific root length (SRL) of the absorptive roots (the first two orders) significantly decreased, while root abundance (root biomass and root length density) and root tissue density (RTD) significantly increased. Fifty-four percent of the variation in the absorptive root traits could be explained by the soil N concentration and the biomass and diversity of the understorey vegetation. Conversely, transport root (third- and higher-order) traits did not vary significantly among different thinning intensities. The covariation of absorptive root traits across thinning intensities regarding two dimensions was as follows: the first dimension (46% of the total variation) represented changes in root abundance and chemical traits (related to RCC, RNC), belonging to an extensive foraging strategy; the second dimension (41% of the total variation) represented variations in root morphological traits (related to RTD, SRL and SRA), which is an intensive foraging strategy (i.e., root economic spectrum). These results suggested that the absorptive roots of Chinese fir adopt two-dimensional strategies to acclimate to the altered surroundings after thinning.

## Introduction

Thinning is a widely used practice in forest management to develop complex stand structures and compositions for fire and insect resistance^1^ and to improve tree growth and stand regeneration^2–5^. The effects of thinning on the aboveground structure and processes have been widely investigated^2,6,7^. However, the response of belowground processes to thinning is still unclear^8^. Fine roots play a key role in taking up water and nutrients and regulating carbon cycling in forest ecosystems duo to high production, rapid turnover, and high respiration rates^9–11^. Despite these factors, the mechanisms underlying fine root acclimation to forest thinning have yet to be elucidated.

Trees capture soil resources (e.g., nutrients and water) via several strategies. First, trees allocate abundant biomass to fine roots and increase the root length density (RLD, Table 1) in nutrient-poor soil^12^, which is known as an extensive foraging strategy^13^ that requires a greater carbon allocation for root formation^14^. Second, trees with a low C investment affect uptake absorption through root morphological and physiological plasticity^15^, which is known as an intensive foraging strategy^13^. This mechanism, also called the root economics spectrum (RES)^14^, implies a trade-off between resource foraging and resource conservation^14^. For instance, trees with increased specific root length (SRL) and specific root area (SRA) and decreased root tissue density (RTD) had higher soil nutrient uptake capacity under nutrient-rich conditions^16,17^ as well as higher N concentrations in roots in nutrient-rich soils^14^. In addition, fine roots can downregulate their respiration rate for enhanced energy to absorb soil nutrients under dry soil conditions^18^; additionally, the root respiration rate is closely related to the N concentration^19^. Finally, some trees associate with microorganisms for the uptake of soil resources^20^, and changes in root morphological traits are related to the species-specific impact of microorganisms^9,21^. Therefore, we can approach the acclimation mechanisms of fine roots to thinning intensities based on root abundance (e.g., biomass, length, and number of roots) as well as morphological and chemical traits.

**Table 1:** List of root traits measured and their demonstrated foraging mechanisms of roots.

Root traits	Abbreviation	Units	Functional significance	References
Specific root area	SRA	cm^2^ g^−1^	Nutrient foraging strategy (+)	Ostonen et al.^15^
Root tissue density	RTD	g m^−3^	Nutrient foraging strategy (−)	Ostonen et al.^14^
Specific root length	SRL	m g^−1^	Nutrient foraging strategy (+)	Lõhmus et al.^13^
Root N concentration	RNC	%	Nutrient foraging behavior (+)	Ostonen et al.^14^
Root C concentration	RCC	%	Nutrient foraging behavior (+)	Prieto et al.^53^
Root number	RN	10^3^ no m^−2^	Nutrient foraging strategy (+)	Ostonen et al.^14,15^
Root biomass	Biomass	g m^−2^	Nutrient foraging strategy (+)	Ostonen et al.^15^
Root length density	RLD	mm^−2^	Nutrient foraging strategy (+)	Ostonen et al.^14,15^

Symbols (+) and (−) indicate positive and negative relationships between traits and functions, respectively.

Thinning can impact fine root traits through the alteration of biotic and abiotic factors in stands^22^; however, these studies did not provide any consistent conclusions. For example, some studies showed that fine root biomass decreased in thinned plots^23,24^, while others revealed different patterns^25–27^. For morphological traits of the root, thinning increased the RLD, SRL, and SRA^7,25,28^ and decreased the RTD^21^ but had no effects on SRL and RLD^29^. These inconsistent results might be attributed to the thinning intensity and species. For example, fast-growing species have higher SRL and lower RTD than slow-growing species^30^. Additionally, forests with different thinning intensities may differ greatly in environmental conditions (e.g., light and soil nutrients) and understorey species diversity. The findings of some studies demonstrated that plants adopt intensive strategies (e.g., high SRL and SRA and low RTD) to take up resources from nutrient-rich soil^31^, while other studies showed low SRL and high RTD in high-quality soil^32^. Additionally, a recent study detected that root traits were impaired by species diversity compared to soil conditions^32^. Consequently, to determine the variation in fine root traits with thinning intensity, the role of understorey vegetation diversity and soil nutrients should be considered simultaneously.

Furthermore, the effects of thinning on fine root traits in previous studies were mostly based on diameter cut-offs (root diameter < 1 or 2 mm). A recent study showed that roots with a diameter < 1 mm include several root orders with different traits and functions^33^. Typically, absorptive roots of the first two or three orders have higher SRL and N concentrations and lower RTD than the transport roots of higher orders; the former are mainly for water and nutrient absorption, while the latter are primarily for storage and transport^34,35^. Moreover, absorptive roots without secondary development are more sensitive to environments than transport roots with the protection of secondary tissue^36,37^. Therefore, branching orders and functional approaches rather than the diameter approach have been shown to be optimal proxies for root functioning. Recently, foraging strategies have been tested in absorptive roots (e.g., the first orders, the first two orders, or the first three orders) and/or transport roots (the higher orders) along a latitude gradient and among various stands and species^14,37,38^. Another study found that the absorptive root orders increased from the first two orders to the first five orders along an elevation gradient^39^, implying that the absorptive and transport roots may be dependent on environmental factors. Therefore, the adaptation mechanism of fine roots to thinning intensity by integrating the response of branching order and functional group root traits to thinning intensity is effective. However, to the best of our knowledge, no data are available to substantiate this finding.

Chinese fir (*Cunninghamia lanceolata*) is one of the most important native species cultivated in China, with considerable economic and ecological benefits^40^. However, Chinese fir plantations over a large area at a high density during the past resulted in relatively low production and a simple stand structure^41^. Thus, thinning was used to release stand resources and improve tree growth and understorey regeneration. In previous studies, the variations in fine root traits of the branching orders and functional groups (e.g., absorptive roots) were detected separately^8^, and therefore, the different responses between branching order and functional group root traits to thinning intensity are poorly understood. This knowledge gap prevents the effective management of Chinese fir plantations. In the current study, we investigated the functional traits of branching-order roots and two functional groups along a thinning intensity gradient in a 26-year-old Chinese fir stand. Our objectives were to determine the following: (1) how do functional traits of the first five orders and functional groups change with thinning intensity? and (2) what strategies are adopted for absorption roots and transport roots to adapt to thinning?

## Results

### Environmental characteristics vary with thinning intensity

With increasing thinning intensity, the DBH (diameter at breast height), shrub biomass, shrub richness, shrub Shannon index, herbaceous richness, understorey plant species, and soil C:N ratio significantly increased, and the stand density, soil nitrogen concentration and root/shoot ratio of trees significantly decreased (Table 2). In addition, thinning intensity did not exert a significant impact on the understorey biomass (the total biomass of shrubs, herbaceous plants, and litter), herbaceous Shannon index, litter biomass, or soil carbon concentration (*p* > 0.05, Table 2).

**Table 2:** Variation in stand characteristics, diversity and soil conditions along the thinning intensity. Parameters (intercept, slope, adjusted *R*-squared, and *p-*value) of the linear models were showed.

Stand properties and soil conditions	Parameters of the linear models			
	Intercept	Slope	*r*^*2*^	*p* value
Stand density (trees ha^−1^)	3100.22	− 2978.60	0.99	** < 0.0001**
Diameter at the breast high of the tree (DBH, cm)	10.45	8.10	0.79	** < 0.001**
Shrub biomass (g m^−2^)	0.16	2.40	0.76	** < 0.001**
Herbaceous biomass (g m^−2^)	0.48	0.70	0.20	0.08
Litter biomass (g m^−2^)	4.39	− 0.66	− 0.09	0.75
Understory plant biomass (g m^−2^)	5.03	2.43	0.04	0.25
Shrub richness	4.08	7.57	0.63	**0.001**
Shrub Shannon index	0.67	0.25	0.58	**0.003**
Herbaceous richness	3.55	3.86	0.63	**0.001**
Herbaceous Shannon index	0.68	0.19	0.19	0.09
Understory plant richness	7.63	11.43	0.77	** < 0.001**
Root/shoot ratio of the trees	0.77	− 0.66	0.78	** < 0.0001**
Soil carbon concentration (%)	1.80	− 0.24	0.02	0.29
Soil nitrogen concentration (%)	0.22	− 0.07	0.63	**0.001**
Soil C:N ratio	8.06	1.75	0.38	**0.02**
Soil water (%)	22.14	− 2.71	0.16	0.11
Soil temperature (°C)	15.98	0.26	− 0.04	0.48

*P* values in bold are significant at *p* < 0.05.

### Variation of fine root traits with thinning intensity

**Figure 1:**
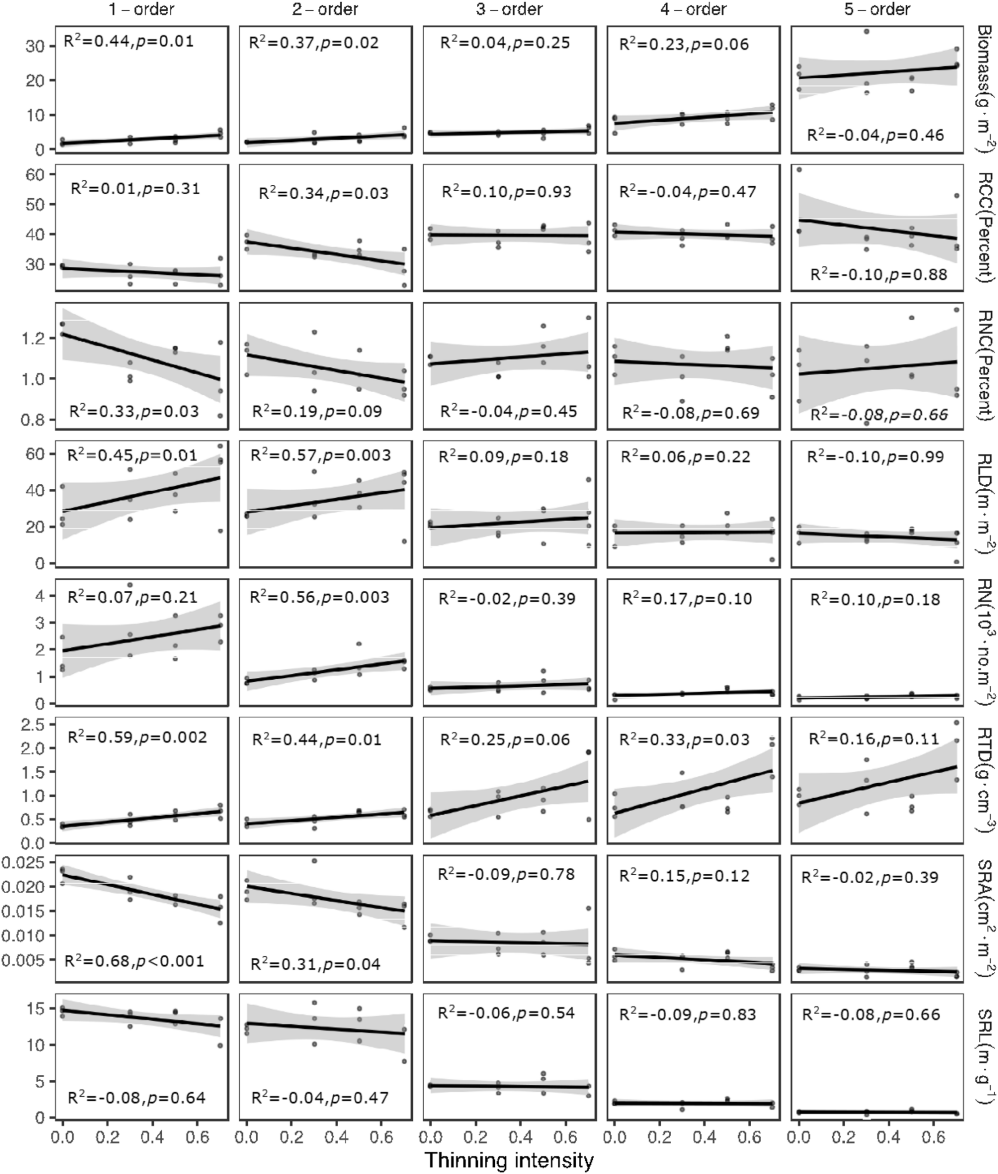
Variation in functional traits of the first five orders roots along the thinning intensity. Adjusted *R*-squared and *p* value of the linear regression models (according to the linear regression analysis) for the first five root orders are showed. Regression lines with 95% confidence intervals (grey shades) show the prediction of linear models.

**Figure 2:**
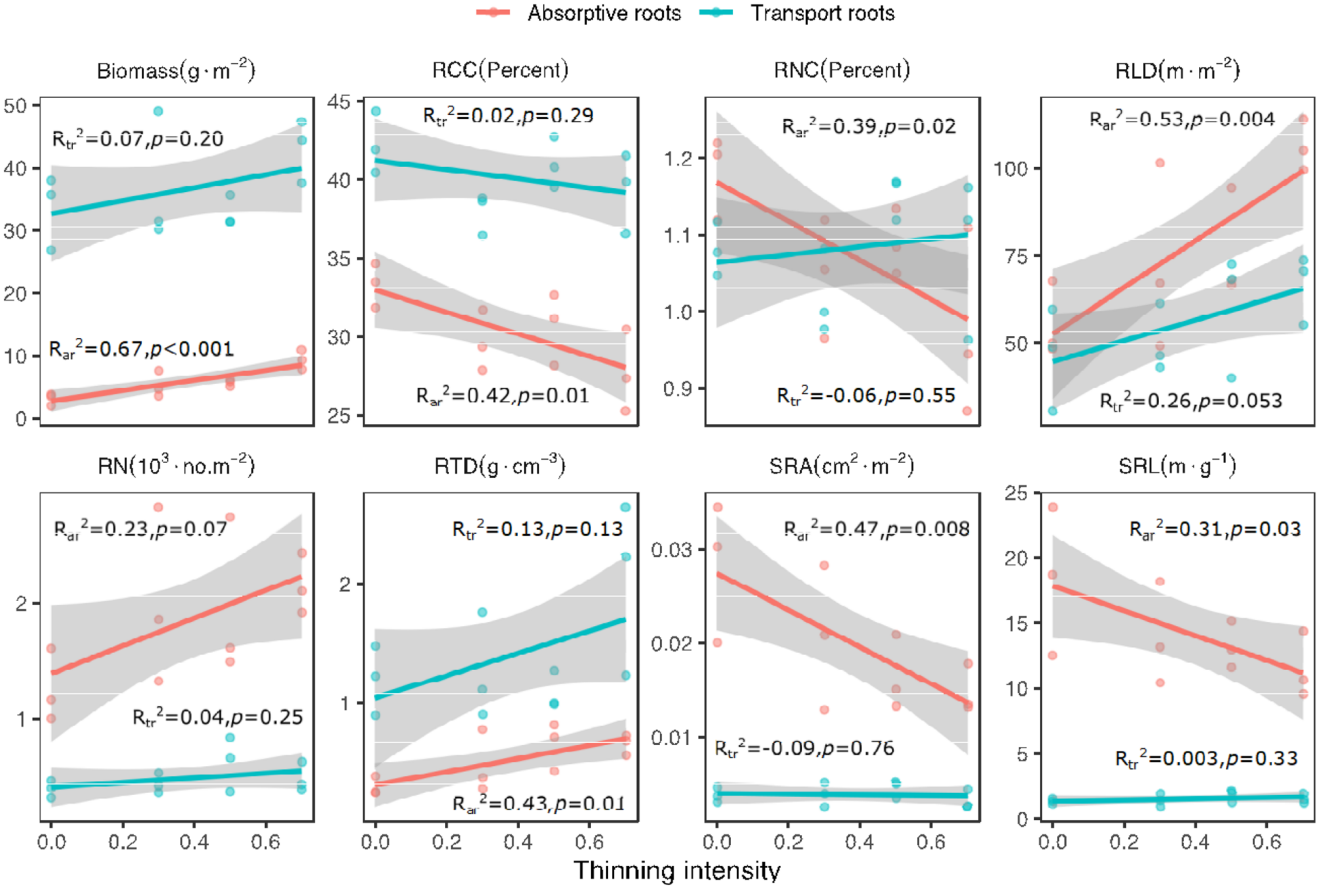
Variation in functional traits of the absorptive roots (red lines and dots) and the transport roots (blue lines and dots) along the thinning intensity. Adjusted R-squared and p-value of the linear regression models (according to the linear regression analysis) for the absorptive roots (R_ar_^2^) and transport roots (R_tr_^2^) are showed. Regression lines with 95% confidence intervals (grey shades) show the prediction of linear models.

With increasing thinning intensity, the biomass, RLD, and RTD of the first two order roots and the second-order root number significantly increased, while the SRA of the first two order roots, N concentration (RNC) of the first-order roots, and C concentration (RCC) of the second-order roots significantly decreased (Table S1, Fig. 1). In contrast, the third- and higher-order root traits did not vary significantly with respect to thinning intensity, except for an increase in the RTD of the fourth-order roots (Table S1, Fig. 1). In the case of the two functional groups, the absorptive root RCC, RNC, SRA, and SRL significantly decreased and root biomass, RLD, and RTD significantly increased with increasing thinning intensity (Table S1, Fig. 2); however, the absorptive root number per unit and the transport root traits did not change significantly with thinning intensity (Table S1, Fig. 1, 2).

### Covariation in fine root traits

**Figure 3:**
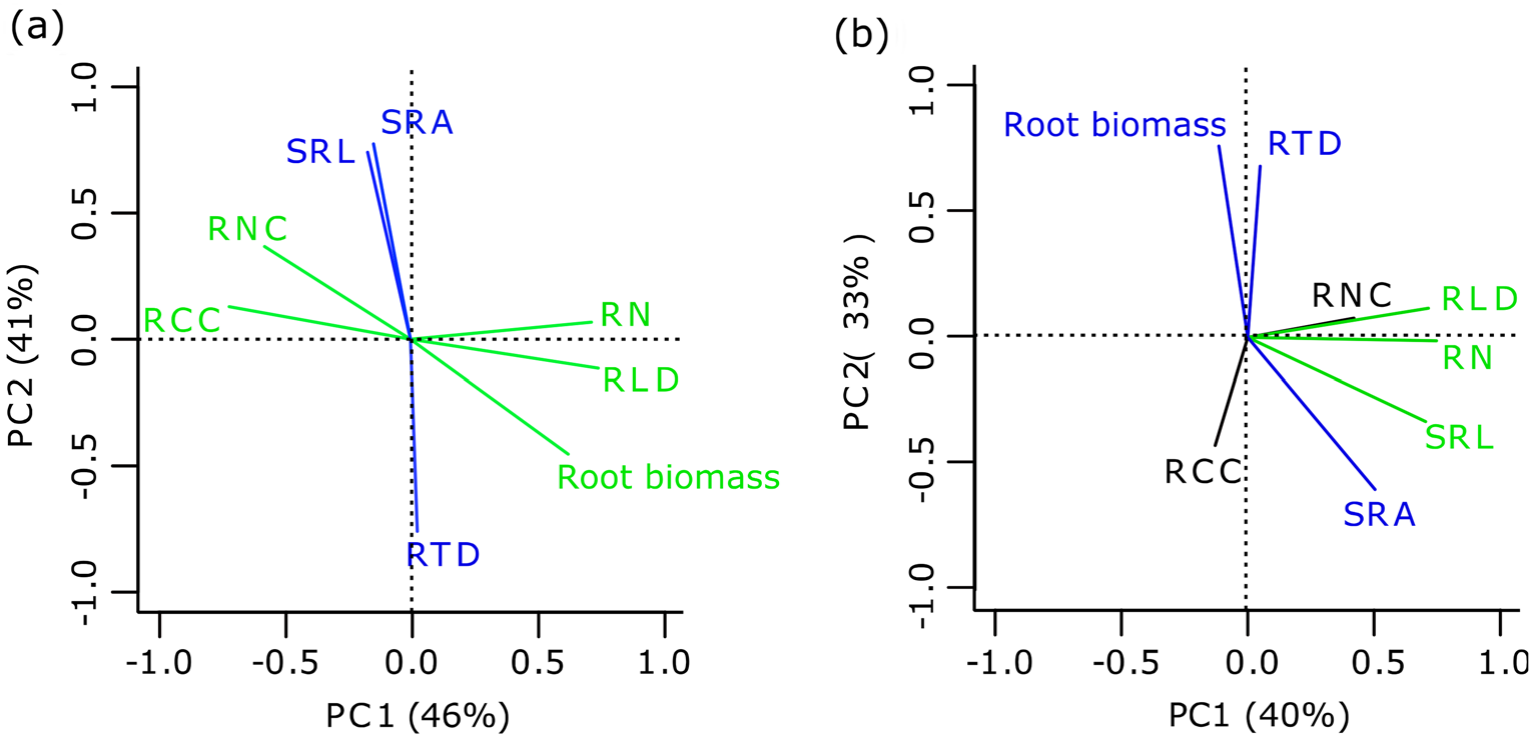
Principal component analysis (PCA) of root functional traits for absorptive roots (**a**) and transport roots (**b**). Traits with green and blue colors were positively related to the first principal component and the second principal component, respectively.

The first two axes of principal component analysis (PCA) based on the eight root traits accounted for a total of 87% and 73% of the variance in the absorptive roots and transport roots, respectively (Fig. 3a, b). The first component (PC1) accounted for 61% and 45% of the variance in the absorptive and transport roots, respectively (Fig. 3a, b), and the second component (PC2) accounted for 26% and 28% of the variance in the absorptive roots and transport roots, respectively (Fig. 3a, b).

For the absorptive roots, PC1 was positively related to RLD, root biomass, and root number and was negatively related to RCC and RNC (Table S2, Fig. 3a), whereas PC2 was positively related to SRL and SRA and negatively related to RTD (Table S2, Fig. 3a). The ordination of thinning intensity along PC1 was opposite to that of higher intensity thinning with high root biomass, RLD, root number, and positive scores and of lower intensity thinning with higher RNN, RCC, and negative scores (*p* = 0.02, *R*^2^ = 0.36). The upward trees on the second axis showed lower intensity thinning with high SRA and SRL and the opposite trend with high RTD (*p* = 0.04, *R*^2^ = 0.30). For transport roots, PC1 was negatively linked to RLD, SRL, and root number, whereas PC2 was positively linked to root biomass and RTD and negatively related to SRA (Table S2, Fig. 3b). However, thinning intensity did not exert any significant effects on the scores of the first two axes of the transport root PCA (*p* = 0.11 and *R*^2^ = 0.15 for PC1; *p* = 0.10 and *R*^2^ = 0.17 for PC2).

For the absorptive roots, RCC was positively correlated with RNC and negatively correlated with RLD, root biomass, and root number (Fig. 3a, Table S3). RLD was negatively correlated with RNC and RCC and positively correlated with root biomass and root number (Fig. 3a, Table S3). On the other hand, root biomass was negatively correlated with SRL and SRA and positively correlated with RNC and RCC (Fig. 3a, Table S3). Furthermore, SRL was negatively correlated with RTD and positively correlated with SRA, while a negative correlation was observed between RTD and SRA (Fig. 3a, Table S3). In the case of transport roots, SRA was positively associated with SRL and RN and negatively correlated with RTD (Fig. 3b, Table S3). SRL was positively associated with root number and SRA (Fig. 3b, Table S3). RLD was positively correlated with SRL and root number, and root biomass was positively associated with RTD (Fig. 3b, Table S3).

### Environmental characteristics and fine root traits

**Figure 4:**
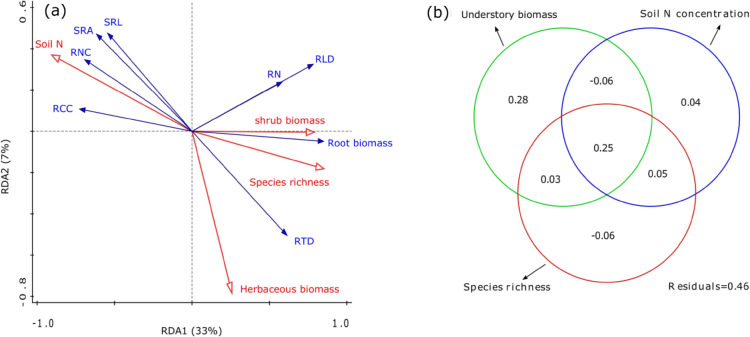
Bio-plot diagram of the redundancy analysis (RDA) for the absorptive root traits (**a**) and the Veen diagram (**b**) of relative contribution of environmental factors (soil condition, stand structure and composition) to variation of absorptive root traits. Lines with empty arrows show environmental factors (red lines), while lines with solid arrows indicate root traits (blue lines).

According to the results of redundancy analysis (RDA), the variation of the absorptive root traits could be ascribed to the environmental factors, but no significant effects were detected on the transport root traits (*p* > 0.05, data not shown) (Fig. 4a). For the absorptive roots, soil N concentration and understorey vegetation biomass and richness accounted for 54% of the variance in fine absorptive traits (*p* = 0.01, *R*^2^ = 0.54, Fig. 4a). Soil N concentration, understorey vegetation biomass, and understorey vegetation richness accounted for 28%, 50%, and 27%, respectively, of the variance in absorptive root traits (Fig. 4b). Additionally, the combined action of the three factors significantly accounted for 25% of the variation in the absorptive root traits (Fig. 4b). Furthermore, absorptive root SRL, SRA, RNC, and RCC were negatively related to the understorey biomass and species and were positively associated with the soil N concentration. In contrast, RLD, root biomass, RTD, and root number were positively linked to understorey biomass and richness and negatively related to soil N concentration (Fig. 4a).

## Discussion

The first two orders of root traits of Chinese fir were more sensitive to thinning intensity than the third and higher orders (Table S1, Fig. 1). This may be related to the different branching-order root structures; the lower-order roots (e.g., the first- and second-order roots) without secondary development are more sensitive to the environment than the higher-order roots with the protection of secondary tissue^34,35^. Consequently, we detected variations in the absorptive (the first two orders) and transport root (the third-order and higher orders) traits with thinning intensity, and the results showed that seven traits of the absorptive roots were significantly changed with thinning intensity, albeit without significant variations in the transport root traits (Table S1, Fig. 2). According to the current study, the absorptive roots of Chinese fir might include the first two orders, which is similar to a previous study^42^. Therefore, a functional approach is preferable to disentangle the effects of thinning intensity on fine root traits and dynamics based on sampling time and labour.

With increasing thinning intensity, absorptive root abundance (RLD and root biomass) and RTD significantly increased, and the SRL, SRA, RCC, and RNC significantly decreased (Fig. 2), which could be partially attributed to the low soil N concentration after thinning (Table 2, Fig. 4a). Previous studies indicated that greater light was available in the forest canopy after thinning, resulting in higher yield by increasing the carbon allocation to root growth and soil resource uptake under nutrient-poor soil conditions^43^. This phenomenon might be related to the optimal partitioning theory, which states that trees allocate more biomass to organs that take up growth-limiting resources^44^. In the present study, soil N decreased with increasing thinning intensity, while absorptive roots were mainly for water and nutrient absorption; thus, Chinese fir allocated more biomass to absorptive roots for foraging soil nutrients in the high-intensity thinned plots. Conversely, light decreased with decreasing thinning intensity, and Chinese fir allocated more biomass to the aboveground leaves to compete for light in the low-intensity thinned plots (Table 2, root/shoot ratio of trees decreased with decreasing thinning intensity). Absorptive root RCC was negatively correlated with the root biomass and RLD (Fig. 3a, Table S3), suggesting that abundant carbohydrates of Chinese fir were transported to absorptive root formation to forage soil nutrients, as described previously^45^. Previous studies also showed that trees allocated more biomass to roots, resulting in lower foraging traits in nutrient-poor soil (e.g., lower RNC, SRA and SRL and higher RTD)^11,32^. In the present study, lower absorptive root chemical traits (RCC, RNC), morphological traits (SRA, SRL), and high RTD were found in the plots with a higher thinning intensity (Fig. 1,2). These changes are partially explained by the lower soil N concentration after thinning (Fig. 4a, b). SRL and RNC are positively related to the root respiration rate^46^. The absorptive root SRL and RNC decreased with increasing thinning intensity (Fig. 3), suggesting that the absorptive root respiration rate may decrease, and consequently, a large amount of energy is utilized for nutrient uptake from the soil^18^. Although the respiration rate of absorptive roots was not measured in the present study, a previous study showed that fertilization increased the respiration rate of absorptive roots^19^. Additionally, fine roots with a lower RNC and a higher RTD exhibited a longer lifespan^47^. Previous studies also found that RTD was higher under nutrient-deficient soil conditions^15^. Consequently, Chinese fir roots with a longer life span may be beneficial for maintaining nutrients in the current study.

Interestingly, based on the present study, the absorptive root traits of Chinese fir were largely affected by understorey vegetation after thinning. The understorey biomass and abundance accounted for 49% of the variance in the absorptive root traits (Fig. 4b), although this topic has not been under intensive focus. A recent study also showed that root traits were impaired by species diversity compared to soil conditions, and new colonizers with foraging traits (e.g., higher SRL and SRA and lower RTD) can compete for soil nutrients from shade trees^48^. In the current study, we did not measure the absorptive root traits of the understorey vegetation, but past studies proved that early-successional species (e.g., herbs) with higher foraging traits (e.g., SRL, RNC, RLD, and lower RTD) could rapidly colonize the stand^49^. Consequently, thinning opened the canopy, light directly shined on the forest floor, nutrients in the forest were released, and then peripheral species grew through seed diffusion into the forest^50^. These pioneers had more activity and higher foraging soil nutrient capacity than the remaining trees with conserved traits (e.g., lower SRL, SRA, RNC, higher RTD)^48^. On the other hand, the effects of understorey vegetation on the absorptive root traits of Chinese fir might be related to interspecific hierarchical competition^51^. Herbs are shallow-rooted vegetation with foraging traits (e.g., higher SRL, RNC), and thus, the capacity of herbs to forage soil resources from the surface soil is higher than that of Chinese fir with conservative root traits (e.g., lower RNC, higher RTD). Consequently, with increasing thinning intensity, Chinese fir may choose to forage nutrients from deep soil to supply aboveground growth, thereby avoiding competition with herbaceous plants for nutrients in the surface soil. Collectively, a trait-based approach may be a better proxy for unravelling the mechanism of understorey regeneration after thinning. Therefore, the variations in functional traits and functional diversity of understorey vegetation need further investigation.

**Figure 5:**
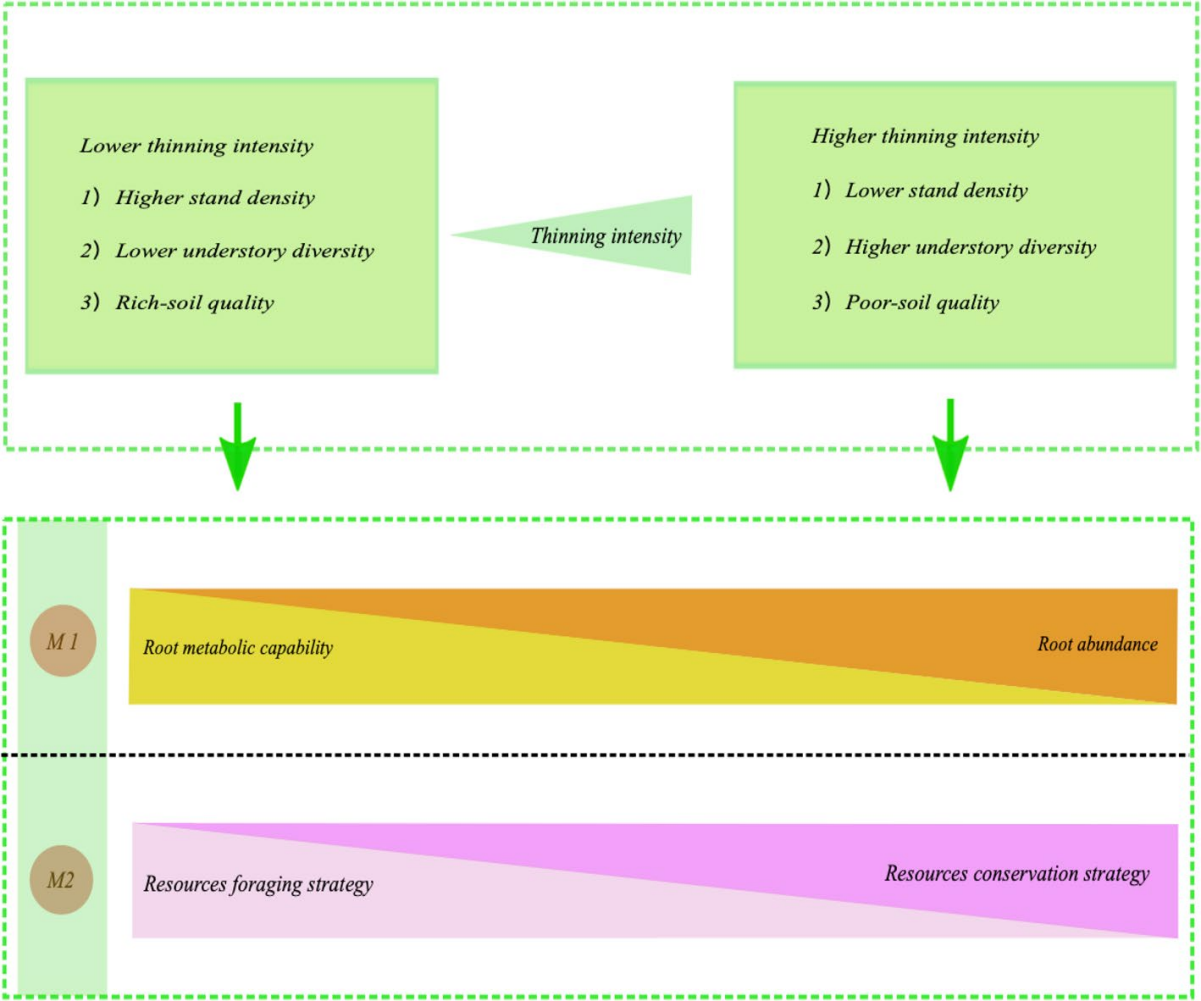
The absorptive root acclimation strategies of Chinese fir along thinning intensity. M1: the trade-off between root abundance and root metabolic capability; M2: the trade-off between resources foraging strategy and resources conservation strategy. The triangles with different colors indicate that the values of indices within triangles increase with increasing width of the triangle.

Individual Chinese fir with high root abundance was loaded on the right of the first axis (PC1) of the absorptive root PCA, whereas Chinese fir with high RCC and RNC showed loading on the left of this axis (Fig. 3a). These results suggested that Chinese fir with higher root abundance aligned in opposition to Chinese fir with higher root chemical traits. With increasing thinning intensity, Chinese fir tended towards higher PC1 values, i.e., higher root abundance (Fig. 3a, Fig. 5), suggesting that this species subsequently adopted an extensive foraging strategy to acclimate to the altered surroundings^14^. Previous studies indicated that the extensive foraging strategy requires a large amount of carbon for root formation^13,15^. Therefore, in the present study, absorptive RCC and RNC were negatively correlated with root biomass and RLD (Fig. 3a, Table S3). Roots with low N concentrations have low root respiration and metabolic rates^46^, and thus, more carbon can be allocated to absorptive roots to forage soil nutrients and water. PC1 of the absorptive root PCA represented a trade-off between the extensive foraging strategy and root metabolic capability (related to RNC). Collectively, with increasing thinning intensity, the absorptive roots of Chinese fir changed from high N and C concentrations and low abundance to low C and N concentrations and high abundance (Fig. 5).

As confirmed by previous studies, acquisition strategies for fine root resources are multidimensional^52^. In the present study, the second axis (PC2) of the absorptive root PCA showed a trade-off of Chinese fir with higher SRL and SRA as opposed to Chinese fir with higher RTD (Fig. 3a, Fig. 5). SRL, SRA, and RTD are the key traits of the root economics spectrum^46,52^, and trees with high SRL and SRA imply effective foraging of resources with a low carbon investment^52–54^, while trees with high RTD imply low effective foraging of resources with a higher carbon investment, representing resource-conserving strategies^55^. Therefore, the second axis of the absorptive root PCA represented the intensive foraging strategy. With increasing thinning intensity, Chinese fir tended towards low PC2 values (high RTD) (Fig. 5), indicating that the absorptive roots changed from the foraging resource strategy (high SRL, SRA) to the conservative resource strategy (high RTD) (Fig. 5). Taken together, the absorptive root traits of Chinese fir used extensive and intensive foraging strategies (or the root economics spectrum) to acclimate to the modified conditions after thinning (Fig. 5).

In contrast to the absorptive root traits, coordinated variations in the transport root traits of Chinese fir along the thinning intensities were not observed. This phenomenon might be related to the different functional and structural properties between the absorptive and transport roots. Transport roots with secondary tissue protection can resist severe disruption of abiotic and biotic factors (e.g., understorey vegetation) and thus be less sensitive to thinning. According to the present study, RES existed in the absorptive roots of the same species, which is in agreement with previous studies that tested the RES in lower-order roots at the species and community levels^8,32,56^. However, some studies showed that there was no RES in the root traits, which could be partially explained by the diverse definitions of fine roots. Therefore, a functional approach may be a better proxy for deciphering the fine root adaptive strategy of Chinese fir after thinning.

## Conclusions

The traits of the first two order roots of Chinese fir were more sensitive to thinning intensity than the third- and higher-order roots, suggesting that the absorptive roots of Chinese fir may include the first two orders. The absorptive root traits of Chinese fir were more sensitive to thinning due to the variations in soil N concentration and understorey regeneration after thinning, whereas transport root traits were less sensitive to thinning. Therefore, the functional traits of understorey vegetation should be detected along a thinning intensity gradient to guide forest management. These results suggested that the absorptive roots of Chinese fir adopt two-dimensional strategies to acclimate to the altered surroundings after thinning.

## Materials and methods

### Study site and plots

The study was conducted in a 26-year-old Chinese fir plantation at the Lishui Tree Farm Research Station (119°01' E, 31°36' N) of Nanjing Forestry University, Jiangsu, China. The site has an altitude of 100 m with a 15° slope oriented towards the south. The soil is a Haplic Luvisol with a maximum depth of 30 cm. The climate in the study area ranges from subtropical to temperate, with a mean annual temperature of 15.5 °C and average annual sunshine duration of 2146 h. The mean annual precipitation is 1005.7 mm, with a frost-free period of 220 days per year.

Twelve 20 × 20 m study plots were randomly selected and subjected to four different thinning treatments, with three replicates (plots) in each treatment. A 10-m-wide buffer zone separated each plot to reduce potential edge effects. Stand thinning was conducted between February and April 2007 by removing all the aerial portions of the trees as follows: 1) all the dead or blight trees were thinned in the plots; 2) trees less than 2 cm in diameter were removed from the largest density stand. Finally, thinning resulted in a stand density of 1020 trees ha^−1^ in the high thinning intensity treatment (70% reduction in stems), 1725 trees ha^−1^ in the moderate intensity treatment (50% reduction in stems), 2450 trees ha^−1^ in the low thinning intensity treatment (30% reduction in stems) and 3495 trees ha^−1^ in the untreated treatment (no thinning, 0%). The main features of the experimental plots in 2011 are shown in Table 2.

### Root sampling, understorey biomass, and diversity

In mid-July 2011, we selected three trees to sample the roots from each plot based on the mean diameter at breast height (DBH) of the stands. One block of soil (20 cm long × 20 cm wide × 20 cm deep) with intact root networks was collected 50 cm from the target tree at a soil depth of 0–20 cm. We identified the roots of Chinese fir based on colour, smell, and attachment to coarse roots. Consequently, 108 blocks (4 treatments × 3 replicates/plots × 3 trees × 3 blocks) were excavated, immediately placed in a cool plastic bag on ice, and transported to the laboratory within hours.

Then, a 1 m × 1 m plot was set around each target tree to investigate the understorey species diversity (e.g., species richness and Shannon index) and to harvest aboveground understorey vegetation (e.g., shrubs and herbs) and litter. The fresh weights of the understorey and litter samples were estimated in situ, and then the samples were dried at 70 °C to a constant weight in the laboratory. The biomass and diversity indices were used as an index to reflect the capacity of the understorey vegetation to compete with trees for soil nutrients. The root/shoot ratio of the trees (Chinese fir) was estimated as coarse root biomass divided by leaf biomass, and the coarse root biomass and leaf biomass were estimated using the biomass relative growth Eq. ^57^.

### Root dissection and morphology assessment

In the laboratory, fine roots were stored in deionized water. The larger intact roots were carefully removed from the soil with a pair of forceps, and the remaining soil on the roots was brushed away and dissected as described by Pregitzer et al. (2002)^58^. The most distal root tips with no branches were defined as the first order, and the roots in which two first-order roots intersected comprised the second order. The remaining branch orders were determined similarly. Then, the order of the roots was scanned, and the captured images were analysed to determine the diameter, length, and number of roots using WinRHIZO Version 2005c (Regent Instrument Inc., Nepean, ON, Canada).

### Root biomass, nitrogen concentration, and carbon concentration

Following scanning, individual roots of a given order were dried at 65 °C for 48 h, weighed (to obtain the biomass of each order), homogenized using a mill, ground, and sieved through a 0.15 mm mesh. RNC and RCC were analysed using an elemental analyser (Vario EL III, Germany).

### Assessment of root traits

SRL (m/g) was estimated as root length divided by root biomass, RTD (g/m^3^) was estimated as the ratio of root biomass to its volume assuming that the root was a cylinder, and SRA (cm^2^/g) was estimated as SRA divided by root biomass. Moreover, the RLD (m/m^2^) was determined by dividing the total root length by the area of the sampling block.

We classified the absorptive roots (the first two orders) and transport roots (the third and higher orders) of Chinese fir according to a previous study^8^ and our results of the effects of thinning intensities on branching-order root traits (Fig. 2). Absorptive RNC and RCC represented the mean of the first two orders of roots, and transport RCC and RNC were the mean of the third- and higher-order roots. Absorptive root biomass, root length, and number of absorptive roots represented the sum of the first two orders, and the transport root biomass, length, and number were the sum of the third- and higher-order roots.

### Soil water, temperature, N and C concentrations

We used five soil augers (diameter 4.5 cm) for each plot to withdraw samples at a soil depth of 0–20 cm near each point of the root area. Then, we combined the five soil samples to generate a single composite fresh sample. Subsequently, the gravel and roots were removed carefully, after which the soil samples were air-dried in the shade and milled to pass through a 0.15 mm sieve for C and N analysis using an elemental analyser. Subsequently, the soil temperature and water content were recorded at a depth of 20 cm at the time of sampling using TDR (JA36TDR-3A, SPT, USA).

### Data analysis

Variations in root traits, soil properties, stand structure, and composition indices with thinning intensity were tested using simple linear regression models with thinning intensity as the fixed factor. The residuals of the models were assessed for normality (Shapiro–Wilk test), homogeneity (Bartlett test), and independence, and variables were log-transformed if necessary^59^. To detect the main dimensions of the root traits, we performed principal component analysis (PCA) to test the distribution pattern of the absorptive and transport roots separately based on eight traits (root biomass, RLD, SRL, SRA, RN, RCC, RNC, and RTD). Pearson’s correlation coefficient was used to evaluate the correlations between the root traits. In the case of each group of roots, parallel analysis suggested that the first two components explained the variation among the root traits, and consequently, the variations in the first two component scores (PC1 and PC2) of the PCA of each group of roots with thinning intensity were also determined using linear regression models. The residuals of the models were assessed for normality, homogeneity, and independence.

To unravel the reasons for changes in the root traits, redundancy analysis (RDA) was performed with soil conditions (soil N concentration, soil C concentration, soil CN ratio), understorey structures (shrub biomass, herbaceous biomass, DBH of Chinese fir), and understorey composition indices (shrub and herbaceous diversity indices) as explanatory variables. In addition, the relative contributions of the soil conditions, stand composition, and structural factors to the variations in root traits were evaluated by the Venn diagram package in R (3.5.3). Then, a forward-selecting RDA was conducted, and the significance (*p* < 0.05) of the RDA results was assessed using a permutation test. All statistical analyses were conducted using R software^59^.

### Statements

All the experiments on plants were carried out in accordance with guidelines of Tongren University.

## Supplementary Information

Supplementary Information.
