# Supplementary material for: Variation in fine root traits with thinning intensity in a Chinese fir plantation insights from branching order and functional groups
Source: Sci Rep. 2021 Nov 22;11:22710. doi: 10.1038/s41598-021-02206-1 (PMC8608833; doi:10.1038/s41598-021-02206-1)
Supplement: Supplementary file 1 — Supplementary Information. [file 41598_2021_2206_MOESM1_ESM.pdf]

Variation in fine root traits with thinning intensity in a Chinese fir plantation insights from branching order and functional groups

Zuhua Wang[1*], Min Liu[1], Fen Chen[1] & Haibo Li[2]

[1] College of A&F Engineering and planning, Tongren University, Tongren, Guizhou 554300, China

[2] National Nature Reserve Administration of the Fanjing mountain, Tongren, Guizhou 554300, China

[*] Correspondence: E-mail: zuhua131666@126.com

Table S1 Variation in functional traits of the branching-order roots and the functional-group roots along the thinning intensity. The intercept, slope, adjusted R-squared and p-value of the linear regression models (according to the linear regression analysis) are showed. *P* values in bold are significant at $p<0.05$.

Traits	Parameters	1-order	2-order	3-order	4-order	5-order	Absorptive roots	Transport roots
Biomass (g/m^2)	intercept	1.76	0.71	4.49	7.59	20.68	2.88	32.75
	slope	3.38	1.00	1.30	4.44	4.59	8.14	10.34
	r^2	0.44	0.37	0.04	0.23	-0.04	0.67	0.07
	p-value	**0.01**	**0.02**	0.25	0.06	0.46	**<0.001**	0.20
RLD (m/m^2)	intercept	26.53	3.27	18.50	15.43	15.49	52.68	44.74
	slope	37.89	0.82	13.77	7.21	0.04	66.75	29.86
	r^2	0.45	0.57	0.09	0.06	-0.10	0.53	0.26
	p-value	**0.01**	**0.003**	0.18	0.22	0.99	**0.004**	0.053
RN (10^3 no./m^2)	intercept	1.96	-0.19	0.57	0.30	0.21	1.39	0.41
	slope	1.32	0.93	0.24	0.22	0.12	1.20	0.20
	r^2	0.07	0.56	-0.02	0.17	0.10	0.23	0.04
	p-value	0.21	**0.003**	0.39	0.10	0.18	0.07	0.25
SRL (m/g)	intercept	14.44	12.97	1.43	2.02	0.77	17.85	1.38
	slope	-1.02	-0.87	0.23	-0.10	-0.11	-9.48	0.45
	r^2	-0.08	-0.04	-0.06	-0.09	-0.08	0.31	0.003
	p-value	0.64	0.47	0.54	0.83	0.66	**0.03**	0.33
SRA (cm^2/m^2)	intercept	0.22	-3.91	0.009	0.006	0.003	0.03	0.004
	slope	-0.01	-0.43	-0.001	-0.003	-0.001	-0.02	-0.0004
	r^2	0.68	0.31	-0.09	0.15	-0.02	0.47	-0.09
	p-value	**<0.001**	**0.04**	0.78	0.12	0.39	**0.008**	0.76
RTD (g/cm^3)	intercept	0.36	0.41	0.58	0.63	0.85	0.32	1.05
	slope	0.44	0.34	1.03	1.30	1.10	0.56	0.94
	r^2	0.59	0.44	0.25	0.33	0.16	0.43	0.13
	p-value	**0.002**	**0.01**	0.06	**0.03**	0.11	**0.01**	0.13
RCC (%)	intercept	28.55	37.46	39.74	40.70	-0.10	33.01	41.25
	slope	-3.52	-10.62	-0.34	-2.03	-0.13	-7.07	-2.92
	r^2	0.01	0.34	-0.10	-0.04	-0.10	0.42	0.02
	p-value	0.31	**0.03**	0.93	0.47	0.88	**0.01**	0.29
RNC (%)	intercept	1.22	1.12	1.07	1.09	1.02	1.17	1.06
	slope	-0.32	-0.19	0.08	-0.05	0.09	-0.26	0.05
	r^2	0.33	0.19	-0.04	-0.08	-0.08	0.39	-0.06
	p-value	**0.03**	0.09	0.45	0.69	0.66	**0.02**	0.55

Table S2 Fine root trait loadings on the two axes of principal component analyses (PCA) for absorptive roots and transport roots. Loadings in bold indicate the significance of the functional traits in PCA ($p < 0.05$).

Functional traits	Absorptive roots		Transport roots	
	PC1	PC2	PC1	PC2
RCC	**-0.90**	0.16	-0.16	-0.53
RNC	**-0.72**	0.46	0.52	0.10
RLD	**0.93**	-0.14	**0.89**	0.14
RB	**0.78**	-0.57	0.06	**0.85**
SRL	-0.21	**0.93**	**0.88**	-0.41
RN	**0.90**	0.08	**0.93**	-0.02
RTD	0.03	**-0.95**	-0.14	**0.95**
SRA	-0.18	**0.97**	0.63	**-0.75**

Table S3 Pearson's correlation coefficients of root functional traits in absorptive roots (lower diagonal, N=12) and transport roots (upper diagonal, N=12); ** and *: correlations were significant at *p*=0.01 and *p*=0.05, respectively.

	RCC	RNC	RLD	RB	SRL	RN	RTD	SRA
RCC		0.03	-0.06	-0.22	0.03	-0.18	-0.42	0.23
RNC	0.84**		0.26	-0.05	0.29	0.54	0.09	0.16
RLD	-0.74*	-0.61*		0.33	0.78**	0.73*	0.00	0.48
RB	-0.74*	-0.81**	0.86**		-0.31	-0.03	0.71*	-0.55
SRL	0.30	0.51	-0.34	-0.70*		0.79**	-0.50	0.87**
RN	-0.70*	-0.45	0.86**	0.60*	-0.17		-0.14	0.57*
RTD	-0.21	-0.46	0.17	0.53	-0.80**	-0.03		-0.84**
SRA	0.29	0.51	-0.33	-0.68*	0.96**	-0.15	-0.92**	
